# From robots to chatbots: unveiling the dynamics of human-AI interaction

**DOI:** 10.3389/fpsyg.2025.1569277

**Published:** 2025-04-09

**Authors:** Albert Łukasik, Arkadiusz Gut

**Affiliations:** ^1^Department of Cognitive Science, Doctoral School of Social Sciences, Nicolaus Copernicus University in Toruń, Toruń, Poland; ^2^Department of Cognitive Science, Faculty of Philosophy and Social Sciences, Nicolaus Copernicus University in Toruń, Toruń, Poland

**Keywords:** human-robot interaction, theory of mind, social cognition, Chatbot, virtual agent

## Abstract

The rapid integration of artificial agents—robots, avatars, and chatbots—into human social life necessitates a deeper understanding of human-AI interactions and their impact on social interaction. Artificial agents have become integral across various domains, including healthcare, education, and entertainment, offering enhanced efficiency, personalization, and emotional connectivity. However, their effectiveness in providing successful social interaction is influenced by various factors that impact both their reception and human responses during interaction. The present article explores how different forms of these agents influence processes essential for social interaction, such as attributing mental states and intentions and shaping emotions. The goal of this paper is to analyze the roles that artificial agents can and cannot assume in social environments, the stances humans adopt toward them, and the dynamics of human-artificial agent interactions. Key factors associated with the artificial agent’s design such as physical appearance, adaptability to human behavior, user beliefs and knowledge, transparency of social cues, and the uncanny valley phenomenon have been selected as factors that significant influence social interaction in AI contexts.

## 1 Introduction

The discourse surrounding artificial agents has undergone a radical transformation in recent years. No longer confined to roles in automobile factories, operating rooms, as opponents in chess games, or as translators, artificial agents are now being introduced as “someone” into psychotherapeutic spaces, often acting as motivators for undertaking new challenges or achieving new life goals (Bhargava et al., 2021).

By integrating artificial agents into these intimate forms of social interaction, we are witnessing claims that these entities can build relationships almost akin to those established between human therapists and their patients. Examples like Conversational AI Interfaces (CAIs)—the technology that allows computers to understand and react to human input to create a dialog—demonstrate the emerging belief in the emotional sensitivity of robots and their ability to reflect and assume the role of a coach or mentor (Sedlakova and Trachsel, 2022). This development marks a significant change in the field of artificial intelligence.

The above advancements compel us to delve deeper into why we, as humans, are beginning to treat artificial agents almost as one of our own—assigning them roles of therapists, psychologists, colleagues, and caretakers of our emotional and mental well-being. The question of what leads us to attribute mental states and a form of mental life to artificial agents is no longer purely theoretical or philosophical. It has become a central and pervasive issue concerning the status of artificial agents in our social lives. This deeper look into current human-artificial agent interaction could be divided into three areas that are likely to become even more important in studying social interaction in the years to come:

- roles that can and cannot be ascribed to artificial agents in our social environment,
- stances that humans can take toward artificial agents,
- dynamics of the human-artificial agent interaction.

To put these claims into context, let us consider an example: social robots that are physically embodied and designed to interact and communicate with humans in a social context. They engage in activities like companionship, education, and assistance by interpreting human emotions and responding appropriately (Breazeal, 2003; Vishwakarma et al., 2024). However, these types of social interaction are not limited to situations where the artificial agent is physically present. AI-driven programs can simulate human conversation as an online chat, as is the case with conversational chatbots. They are commonly used in customer service, providing automated responses to user inquiries, assisting with troubleshooting, or guiding users through processes in a conversational manner (Shawar and Atwell, 2007; Irfan et al., 2024). However, recently they have also gained both public and academic interest as potential colleagues that someone may speak to during hard times (Sallam, 2023). We can also distinguish AI Companions which are forms of artificial intelligence designed to offer personalized interactions and companionship to users. They combine elements of virtual agents, conversational chatbots, and sometimes social robots to create a supportive and engaging experience, often aimed at enhancing well-being and providing emotional support (Fong et al., 2003). Moreover, it has been established that models like ChatGPT pass the Theory of Mind (ToM; Strachan et al., 2023; Kosinski, 2023),^1^ as well as the Turing Test (Mei et al., 2024)^2^ and Faux Pas Test (Shapira et al., 2023),^3^ which serve as supporting evidence of their human-like social cognition. Passing the aforementioned mentalization tests, suggests that the social interactions with artificial agents are embedded in the competent that these agents display.

This emerging landscape highlights the need for comprehensive research into social interactions with artificial agents, particularly concerning their roles in conversation, emotional support, and therapy. It marks a departure from the traditional approaches where artificial intelligence was primarily studied in contexts like industrial automation or informational chatbots (Yang and Hu, 2024). The integration of artificial agents into deeply personal and emotionally significant aspects of human life underscores the urgency for new research perspectives and ethical considerations in the development and deployment of AI technologies.

Firstly, it is imperative to distinguish and compare the various types of artificial agents, including robots, avatars, and conversational chatbots. By exploring this diversity, we aim to determine whether physical appearance, perceived mental features and visualization significantly impact human-AI interactions. Does the human-like appearance of an agent foster a stronger connection, or are their disembodied functions and capabilities more significant in establishing meaningful interactions?

Secondly, we must delve into the psychological reasons that lead humans to want to engage socially with artificial agents. This involves analyzing why we attribute mental states, intentions, and thoughts to entities we know are artificially intelligent—a process known as mind reading or mind perception (Gray et al., 2007; Koban and Banks, 2024). Understanding the cognitive and emotional attributes we assign to these agents can shed light on the depth of our interactions and the potential for artificial agents to fulfill roles traditionally occupied by humans.

To address these topics, we will try to answer the following questions:

- How is social interaction impacted by different forms of artificial agents (humanoid robots, virtual avatars, chatbots)?
- Does the physical appearance or visualization of an artificial agent significantly influence the process of social interaction?
- What makes humans engage in social interaction with artificial agents, attribute mental states, interpret behavior, and ascribe emotions?

Therefore, in this paper we will first review the different forms of artificial agents in existence today, discussing the common features as well as differences that might play a role in their socio-cognitive capacities. Secondly, we will examine the factors that are important for social cognition such as emotions, context, and. The last segment of the discussion will the feeling of eeriness caused by interacting with semi-human agents known as the uncanny valley (Mori et al., 2012) and how it influence social interaction between humans and artificial agents impacting the mind attribution and accompanying emotions.

## 2 Overview of artificial agents

Artificial Intelligence (AI) has rapidly evolved, impacting healthcare, education, entertainment, and communication as AI systems like robots, chatbots, and virtual avatars enhance efficiency and personalization in daily interactions (Smith et al., 2021). Understanding their cognitive and emotional impact is crucial as these systems are widely used in social interaction. The following section will describe the current research and terminology necessary to understand what kind of social interaction is possible between humans and artificial agents. Each form of AI realizes the intended functions and goals set by engineers and programmers differently. Additionally, each form has different limitations that, from the point of view of human biology and psychology, can hinder the emergence of social cognition (Sandini et al., 2024).

Research shows that AI evokes various cognitive and emotional responses depending on the context and type of artificial agent (Glikson and Woolley, 2020). Chatbots in customer service influence satisfaction and engagement through sophisticated natural language processing (Crolic et al., 2022). Avatars in gaming and virtual reality provide immersive experiences, affecting user perception and interaction (Lee and Nass, 2003; Lee and Ji, 2024). Robots, from simple service bots to advanced humanoids, add complexity to human-robot interaction in healthcare, education, and homes. Human-like androids and virtual avatars elicit strong social and emotional reactions, with their physically enacted social cues influencing user responses. More human-like artificial agents evoke empathy and social bonding (Bickmore et al., 2005; Seitz, 2024). Understanding human reactions to these agents is essential for designing effective, user-friendly AI systems and mitigating negative effects like anxiety (Anisha et al., 2024). The impact of artificial agents on people varies depending on the context, modality (text-based, voice-based, embodied), and social cues, just to name a few. For instance, robots in healthcare provide physical and emotional support, while chatbots in customer service resolve issues efficiently (Bickmore and Gruber, 2010). Assessing these variables is essential to tailor AI systems to specific needs, maximizing benefits and minimizing adverse effects.

AI’s current state offers both opportunities and challenges. Diverse artificial agents affect human interaction in various ways. Continued research in human-artificial agent interaction is crucial for developing beneficial, trustworthy AI systems aligned with human values (Smith et al., 2021). This research is increasingly fragmented due to new algorithms, market developments, and advanced language models like ChatGPT (Kosinski, 2023) and virtual assistants like Replika (Pentina et al., 2023). The debate on AI interaction requires an interdisciplinary approach, understanding technical capabilities, emotions, and social cognition comparisons between humans and artificial agents. It is then essential to distinguish between the various forms of artificial agents because, as it was already mentioned, each of these forms brings unique capabilities, limitations, and psychological impacts to interactions with humans, affecting everything from emotional responses to the attribution of agency and intentionality (Ziemke, 2023).

To provide an overview, artificial agents are categorized in this paper by interaction type, by which they can communicate and work with the users. Key categories include:

1. Physical interaction—robots, designed for direct physical interaction with humans or the environment. This physical contact is important in areas such as geriatric healthcare (in example assisting an older person; Robinson et al., 2014) or psychotherapeutic environment (in example robot-animals that children with special needs may hold; Cano et al., 2021).
2. Virtual interaction—autonomous avatars, existing purely in digital environments. The goal of the autonomous avatars is to assist humans in virtual environments (in example assisting through rehabilitation phase in virtual reality; Abedi et al., 2024) or engage with them to create a sense of immersion in video games (Ramadan and Ramadan, 2025). Although users cannot interact with these avatars in physical sense, by adapting the additional technology like virtual reality, they can experience a sense of social presence (Wang et al., 2024a).
3. Conversational interaction—chatbots, focused on natural language interactions through text or speech. The primary interaction happens via prompt written by a user (in the case of ChatGPT, Claude or Gemini) or voice command (in the case of Siri or Alexa). Today chatbots are used as search engines (Selvi et al., 2024), assistants for writing a code (Casheekar et al., 2024) as well as educational tutors (Akpan et al., 2025).

The categorization displayed above, although not comprehensive, is enough to point out the most general differences between artificial agents within the scope of this review. Additional factors that can be taken to further differentiate robots, avatars, and chatbots will be mentioned in the paper, based on the selected articles. The following sections will explore how these different AI forms engage in social cognition, beginning with robots and androids while indicating that the amount of research that compares various types of AI forms in social cognition is still limited.

## 3 AI design types

As each artificial agent may interact with the human in different ways, thus influencing user’s reception and behavior, it is firstly worth pinpoint what are the main characteristics of each of the design type. Each of the type was designed with respect to different forms of interaction it can provide (virtual, physical, conversational) but also based on the settings it should be used like healthcare or clinical, daily assistance with information or guidance through virtual settings. What should be kept in mind is the fact that, as technology progresses, the clear boundaries between those interaction types are getting blurry. For example some of the robots like Pepper can now be supported with ChatGPT module, which allows them to speak and respond the user’s voice (Bertacchini et al., 2023), while chatbots like Claude (Syamsara and Widiastuty, 2024) or Gemini (Haman et al., 2024) also provide audio-based communication instead of only text-based interaction like it was firstly intended. Similarly, avatars are also getting support to provide more immersive and natural interaction-for example virtual reality technology can create a common space and the sense of social presence (Combe et al., 2024) between humans and avatars can also speak to humans thanks to the implementation of large language models (Rao Hill and Troshani, 2024). Next three sections will provide an overall description of particular types of artificial agents, after which the paper will focus on comparing each design type in the context of social interaction highlighting differences and similarities.

### 3.1 Robots

Robots are machines programmed to perform tasks automatically, commonly utilized in industries such as manufacturing, medicine, and exploration. Some of the forms of the robots like androids are designed to closely resemble humans in appearance and behavior, using advanced artificial intelligence to mimic human interactions (Doncieux et al., 2022). Using androids in research in social sciences enhances immersion and ecological validity, setting a higher standard than traditional robots (MacDorman and Ishiguro, 2006). Presenting robots to various age groups and cultures allows researchers to study social development stages and cultural differences in social cognition and attitudes toward artificial intelligence (Yam et al., 2023). Increased perception of agency and emotionality toward machines can lead to positive attitudes toward AI and improved decision-making when collaborating with androids (Perez-Osorio and Wykowska, 2020). This collaboration often involves scenarios where the robot may suggest answers or agree with the human participant, intuitively granting a sense of autonomy to the artificial agent. In human-robot interaction studies, one of the goals is to replicate human traits within these mechanized agents (Smith et al., 2021). For example, robots like iCub have been used in research to examine how attitudes toward robots influence cooperation (Siri et al., 2022). These findings suggest that, after accounting for sex differences, men considered socio-emotional abilities displayed by the robot, which slowed task completion—indicating social inhibition about the robot. The iCub robot, due to its human-like traits, provides valuable insights into human cognitive and emotional processes (Marchesi et al., 2019).

Not all robots are designed to resemble humans as much as possible. The type of robot’s appearance—humanoid, machine-like, or product-oriented—plays a critical role in shaping user expectations and interactions (Dautenhahn and Saunders, 2011). Humanoid robots, which closely mimic human features, like androids, tend to be perceived as more suitable for social roles, while product-oriented robots, designed primarily for functionality, excel in task-specific environments such as healthcare or service industries. This categorization affects both social engagement and user satisfaction during human-robot interaction (Kwak et al., 2014). As technology advances, the definition of “robot” continues to evolve, and classifications such as android, humanoid, mechanoid, machine-like, and zoomorphic robots, among others, offer various frameworks for differentiation. However, the appearance and functional capabilities of these robots vary greatly even within these categories, influencing how humans perceive and interact with them (Su et al., 2023). Different robot designs, from simple machine-like robots to more complex anthropomorphic designs, are used in distinct contexts like hotels, workplaces, and everyday life. The interaction varies depending on whether the robot is encountered briefly, such as a receptionist robot, or in long-term interactions like serving as a companion or co-workers in ecologically valid environments (Dautenhahn, 2018).

Robots can also be classified by their roles within the interaction, such as assistive robots that help the elderly or disabled, and social robots that engage in peer-like interactions to provide companionship or education (Kachouie et al., 2017). These differentiations highlight how the context and nature of human-robot interaction shape the design and function of artificial agents (Goodrich and Schultz, 2007). For example, in Japan, robots such as Aibo (Joshi et al., 2024), RoBoHoN (Yamazaki et al., 2023), and LOVOT (Tan et al., 2024) are integrated into people’s daily routines, including personal and communal social rituals.

To recap, the design of robots significantly influences user perceptions and interaction. Humanoid robots, which exhibit a close resemblance to humans, tend to be more compatible with social roles, while product-oriented robots are optimized for task-specific applications.

### 3.2 Avatars

Digital entities such as avatars, which exist within virtual environments, present unique opportunities for research and practical applications. Unlike physical robots, avatars as virtual agents offer extensive customization of features but lack a physical form, a crucial aspect of social engagement (Morrison et al., 2010). Research indicates that perceived warmth in virtual agents is negatively associated with fear of technology: individuals who fear technology more tend to attribute more negative emotions to virtual agents and interact with them less (Stein et al., 2020).

As digital representations of users in virtual environments, avatars can be categorized based on their visual fidelity and behavioral characteristics. These digital entities range from simplistic, cartoon-like figures to hyper-realistic humanoids, allowing researchers to manipulate the appearance and behavior of avatars for experimental purposes. Studies on the Proteus effect^4^ have demonstrated that an individual’s behavior can change due to their avatar’s appearance, as more realistic avatars tend to induce behaviors aligned with social expectations (Yee and Bailenson, 2007). Avatars offer the flexibility to control factors like race, gender, and facial expressions, making them useful in studying social dynamics and identity in virtual spaces.

Autonomous avatars, powered by AI rather than human users, offer distinct opportunities for investigating human-AI interactions. In contrast to human-controlled avatars, autonomous avatars operate independently, allowing researchers to regulate social interactions within a virtual environment. These avatars are utilized to analyze perceptions of social cues, trust, and realism. For example, in educational settings, AI-driven avatars can deliver customized instruction, replicate authentic interactions, and alleviate cognitive burdens by offering contextualized learning experiences (Fink et al., 2024). In rehabilitation, AI-powered avatars are employed in virtual therapy sessions to aid in physical and cognitive exercises, providing support to patients in environments that adjust based on their progress (Veras et al., 2023). Autonomous avatars are also used in virtual worlds, such as metaverse,^5^ to replicate lifelike social interactions, making them valuable for research and practical applications in virtual spaces (Wang et al., 2024b).

Developers can program social cues, such as facial expressions and gestures, into avatars, creating controlled experimental environments within virtual spaces (Kyrlitsias and Michael-Grigoriou, 2022). Meanwhile, researchers can influence human-AI interaction in activities like joint problem-solving by manipulating variables that affect social cognition. For instance, in a study involving an ultimatum game, participants were presented with descriptions of AI opponents portrayed as emotional or rational. The results indicated that AI perceived as intentional and emotional received higher fairness ratings and elicited more generous offers (de Melo et al., 2013). The level of cooperation with avatars also hinges on team organization and whether an avatar (or NPC^6^ in the context of video games) is viewed as a tool or a teammate. When AI was regarded as a teammate, participants displayed more emotional investment, employed optimal strategies, exchanged more strategic messages, and expressed greater confidence, trust, perceived similarity, and a sense of community compared to when AI was treated solely as a tool (Waytz et al., 2014).

Taken together, in the case of avatars, like the robots, visual design is pivotal in shaping users’ perceptions of these agents, as highly anthropomorphic representations frequently elicit discomfort. Still, this effect can be easily changed by using proper software. The variability in fidelity among avatars—ranging from simplistic, cartoon-like designs to hyper-realistic humanoids—provides researchers with a unique platform to explore identity construction and social dynamics. The chosen level of reality depends on the function that the avatar should play when engaging with the human, which is the same case when it comes to their behavior. Virtual agents can perform independently and facilitate tailored interactions across diverse applications, including education, therapeutic interventions, and immersive environments such as the metaverse. Such virtual environments provide precise control over social variables, encompassing facial expressions and gestural communication, rendering them particularly suitable for investigations into human-AI interactions. In conclusion, digital agents present substantial advantages in research applications due to their inherent flexibility and capability to simulate lifelike interactions. However, their psychological and ethical effects, especially concerning user dependency and their influence on cognitive and emotional well-being (like in the case of Replika), warrant thorough examination in both their development and implementation.

### 3.3 Chatbots

Chatbots, also known as conversational agents, have become essential in various fields, including healthcare, social cognition studies, and customer service (de Cock et al., 2020). These AI-powered systems mimic human conversation and are widely utilized to handle user queries, offer assistance, and facilitate issue resolution across diverse industries. Research on chatbots has concentrated on their capacity to improve user satisfaction, trust, and engagement, while also addressing the emotional and cognitive aspects of their interactions (Ruane, 2019). As conversational agents, they utilize machine learning^7^ and natural language processing^8^ to engage with users through speech or text. Their widespread presence has a significant impact on fields like computer games (Lim and Reeves, 2010; Safadi et al., 2015), healthcare (de Cock et al., 2020), and social cognition studies (Lee et al., 2021). Several notable examples of chatbots and large language models include OpenAI’s ChatGPT (Dao, 2023). Google’s Gemini (AlGhozali and Mukminatun, 2024), Anthropic’s Claude (Berrezueta-Guzman et al., 2024) and, most recently, Le Chat^9^ and DeepSeek.^10^

Chatbots might serve different operational goals, from supporting users in simple, repetitive tasks to engaging in conversation and providing guidance as well as companionship. The adaptability and broad applicability of chatbots make them indispensable tools for various sectors. Their ability to personalize interactions and evolve through learning enhances user satisfaction and broadens their potential for both practical and research-oriented applications.

## 4 Chosen aspects of social cognition in human-artificial agent interaction

To better understand the mechanisms of social cognition between humans and artificial agents, it’s essential to firstly investigate what is the process of social cognition as a whole. Storbeck and Clore (2007) emphasized the deep interconnection between cognition and emotion, providing a critical lens for understanding social cognition in human-AI interaction. Their research challenged traditional views that treat cognition and emotion as separate processes and instead argued that they dynamically shape each other (Storbeck and Clore, 2007). Positive emotions can enhance cognitive flexibility and creativity, while negative emotions can sharpen focus and analytical thinking. This interplay is particularly relevant to AI interactions, where human users evaluate artificial agents both rationally and emotionally. The uncanny valley effect—a phenomenon where near-human AI elicits discomfort—can be explained through this lens. When users cognitively assess an artificial agent that appears almost but not entirely human, subtle inconsistencies may trigger negative emotional responses. This response is heightened when AI exhibits near-human appearance but lacks natural emotional expression or movement, disrupting users’ expectations and leading to a sense of unease. Understanding the cognitive-emotional interaction is essential for improving AI design, ensuring that artificial agents elicit trust and engagement rather than discomfort and rejection.

Beyond cognition-emotion interdependence, Levine et al. (1993) highlight the fundamental role of social interactions in shaping cognitive processes. Their research emphasizes that cognition is not an isolated function but one deeply embedded within social contexts, where knowledge and understanding are collectively constructed. Socially shared cognition influences learning, decision-making, and problem-solving, reinforcing the idea that intelligence is not solely an individual trait but often a collaborative process. Communication and language play a pivotal role in this shared cognition, serving as mechanisms for aligning mental models and negotiating meanings. Furthermore, motivation is closely tied to cognition, with social interactions driving cognitive engagement, attention, and information retention. Cultural frameworks and social norms further shape cognitive interpretations and expectations, impacting how people interact with others—including artificial agents. This might suggests that for artificial agents to be effective social partners, they must align with human social norms and expectations, facilitating interactions that feel natural, trustworthy, and meaningful.

A foundational study by Nass et al. (1994) demonstrated that humans instinctively apply social cognition concepts to artificial agents, treating computers and other AI-driven systems as social actors. Their research revealed that people unconsciously follow social norms, such as politeness and reciprocity, when interacting with computers. This phenomenon, later expanded into the Media Equation Theory (Reeves and Nass, 1996), established that people respond to artificial agents as they would to humans. The study also found that factors like similarity and ingroup bias influence user attitudes toward AI, aligning with existing social cognition theories. These findings were instrumental in shaping the field of human-robot interaction (HRI), providing early evidence that robots and other artificial agents could be studied within the framework of social cognition. Current studies expand on those findings while adding other key concepts and factors that influence human-artificial agent interaction in the response to fast-evolving forms of AI.

Further and one of the most recent research by Guingrich and Graziano (2024) builds upon these foundational studies by analyzing how AI features such as appearance, voice, and behavior contribute to mind perception and social interaction outcomes. Their study suggests that human-like appearances in AI, particularly in social robots and avatars, increase the likelihood of users attributing consciousness to these entities. This tendency aligns with social cognition theories that explain how humans ascribe agency to non-human entities exhibiting human-like traits (Thellman et al., 2022). Similarly, AI systems equipped with natural, human-like voices enhance perceptions of intelligence and social presence, making interactions feel more natural. Adaptive behaviors, including context-aware responses and emotional sensitivity, further reinforce the perception of AI as conscious and socially competent. Additionally, previous studies by the same authors (Guingrich and Graziano, 2023) on chatbots, particularly companion chatbots like Replika, have examined how mind perception in AI relates to social outcomes. The research indicates that users who attribute higher levels of consciousness and human-like qualities to Replika report significant social health benefits. Contrary to concerns that AI companionship might replace human interactions and negatively impact social well-being, findings suggest that users of Replika experience improved emotional support and a sense of social connection. These effects align with broader social cognition literature, which suggests that perceived agency and intentionality in AI enhance relational and emotional interactions. By fostering trust and emotional attachment, chatbots like Replika contribute positively to users’ social well-being, demonstrating the expanding role of AI as a companion and support system.

Another key concepts such as trust, attachment, empathy, acceptance, and disclosure are also extensively studied in fields regarding the social process involved in interaction with artificial agents (Hancock et al., 2020). Artificial agents designed with social cues and behaviors can evoke emotional responses and foster social bonds, increasing their acceptability and effectiveness in roles such as education, healthcare, and companionship (Belpaeme et al., 2018). Understanding how humans process and apply information about social beings is essential to social cognition research, which both informs and is informed by the development of social robotics (Wiese et al., 2017; Broadbent, 2017; Złotowski et al., 2015). As some of the research suggests, human-like properties and attitudes toward artificial intelligence depend on three main factors: the framework, the robot’s social behavior, and the interaction environment (Wallkotter et al., 2020). The framework involves personal experiences and knowledge that influence perceptions in new situations. The robot’s social behavior includes human interaction patterns like nodding and commenting. The environment encompasses the study setting, whether in a laboratory or natural conditions like streets or hospitals. These factors affect perceptions of AI as intentional entities, often assessed through questionnaires like the Godspeed (Bartneck, 2023) and Mind Perception Questionnaires. Findings indicate that robots exhibiting social gestures are perceived as more social. These findings however can also be translated into avatars since they can be programmed with specific animations and responses in a virtual environment (Starke et al., 2020).

Other studies concerning the aspect of communication may suggest that this part of social cognition is better tailored in chatbots (Ali H. et al., 2024), especially the version equipped with the natural-like voice (Hwang et al., 2024) which additionally underscores choosing the proper form of AI for investigating a particular aspect of human-AI interaction. As a common point between different forms of AI, cognition, and emotion are inseparable processes in human interaction especially in social interaction. Positive emotions like happiness, trust, and safety—or negative ones like sadness, anger, and uncanniness—play critical roles, especially with service robots (Chuah and Yu, 2021). Implementing complex emotional reactions in artificial agents can benefit joint tasks, test acceptance of new technologies, and facilitate the introduction of robots and androids in healthcare settings (Indurkhya, 2023) but also implementing chatbots as a part of mental health prevention (Sarkar et al., 2023).

Therefore, various forms of artificial agents—including robots, androids, avatars, and chatbots—offer both common and different variables that can be adjusted based on research hypotheses and the type of social interaction being studied (Glikson and Woolley, 2020). To give an example, some studies focus on cooperation between humans and artificial agents, while others explore competition and collaboration (Shin and Kim, 2020). There are certain themes to be most central when considering the issue of AI-human social interaction:

- embodiment—physically present, virtual, text-based agents (Memarian and Doleck, 2024),
- emotional dynamics—emotional expression manifested by the agent, reactions to the emotions manifested by the human but also hidden expressions both via gestures and the tone of the written statement (Krueger and Roberts, 2024),
- social bonds—the degree to which the user can relate to the agent (Zhang and Rau, 2023),
- expectations—the relation between predicted agent’s behavior and it is actual response with the emphasis on prediction error present in uncanny valley effect (Vaitonytė et al., 2023),
- other aspects such as the adaptability of the agent’s behavior, and humans’ beliefs about artificial intelligence and usability (described further in the paper).

In the following subsections, each type of artificial agent id discussed, concerning the above themes.

### 4.1 Factors influencing social cognition in AI-human interaction

Designing AI capable of engaging users on emotional and cognitive levels requires consideration of a wide range of factors. For example, the impact of bodily expression—including biological versus mechanical movement, gesture presence, and movement speed—and the agent’s ability to recognize emotions during interactions are significant (Hortensius et al., 2018). These factors can be applied to physical agents like robots by providing them with a proper set of joints and virtual agents like avatars with appropriate animation.^11^ However, this will not apply to the chatbots since they do not possess any form of visual representation besides generated text or speech. This type of representation excludes the use of chatbots in some of the studies from the neuroscience field like measuring the activity of the Action Observation Network involving premotor, temporal, and medial temporal areas and the Person Perception Network involving the temporoparietal sulcus (Henschel et al., 2020), as well as studies regarding the mirror neuron system (Gazzola et al., 2007). On the other hand, the current state of chatbots allows researchers to examine neural activity during a conversation with an artificial agent as large language models become more advanced and faster in responding to humans with better accuracy (Kedia et al., 2024). But at the same time variables such as the agent’s anthropomorphism, scale (big or small size), bimanual manipulation, and locomotion are also important for developing effective human-robot interactions (Kerzel et al., 2017) but cannot be applied to text or speech-based chatbots. Some of the current research showed that brain activation regarding the pragmatics is lower during human-robot interaction compared to human-human interaction because of the lack of natural human speech within robots (Torubarova et al., 2023), which creates an opportunity to replicate such studies with new forms of AI. Additionally, research on the temporo-parietal junction and its role in Theory of Mind, suggests that the this region is selectively activated when individuals infer others’ beliefs and intentions, distinguishing it from adjacent brain regions involved in perceiving physical characteristics of human-like entities (Saxe and Kanwisher, 2013). If the TPJ is central to how humans infer and predict others’ thoughts and intentions, it raises important questions about AI design. AI systems that mimic human social behavior without genuine mental states may fail to engage the TPJ in the same way human interactions do, leading to differences in trust, acceptance, and engagement. Understanding this distinction can help refine AI models to better align with the cognitive processes underlying human social interaction.

Investigations into whether interactions between humans and robots differ from human-human interactions in establishing social bonds during conversation have shown that human-robot interactions result in decreased activity in the fusiform gyrus, an area associated with face perception (Spatola and Chaminade, 2022). While increased activity in the posterior cingulate cortex, associated with social cognition, is observed during longer interactions with humans, no such effect is seen during interactions with robots. This suggests that robots are not considered valid social partners. Still, it also creates another opportunity to test this hypothesis in virtual avatars whose faces can be easily adjusted to the environment, role, and type of planned social interaction. It is also easier to monitor longer interactions in virtual reality compared to the lab settings with robots. Some of the studies (Mustafa et al., 2017) already compared different types of artificial agents while evaluating the N400 component using EEG but using stimuli consisting of static pictures with different levels of realism among robots, androids, and avatars with no actual interaction. Future studies focusing on the perception of faces should focus on actual interaction with setup considering robots, androids, and avatars on both screens and in virtual reality.

As already mentioned, besides faces, chatbots also lack bodies, which also excludes them from use in research investigating the perception of social-relevant stimuli like body parts and gaze cues. As the robot’s head may attract the most attention the fix duration may also depend on the emotional expression (Li et al., 2022) but the studies investigating gaze and fixation toward relevant stimuli mainly focus on physically present agents while the current state of virtual reality already allows to gather eye-tracking data, making the use of virtual avatars and their embodiment (Adhanom et al., 2023). Physiological responses are already being studied when interacting with virtual agents but this interaction mainly happens through the screen (Teubner et al., 2015) rather than in a virtual reality when both humans and agents are socially present. Going further, virtual presence separates avatars and chatbots from robots and androids since the first two forms are easier to implement because of their lack of physical bodies. This creates an opportunity to investigate avatars and chatbots in settings regarding cooperation and competition in areas like video games (Possler et al., 2022), dedicated virtual reality settings (Walker et al., 2023), and simulations (Murnane et al., 2021). Cooperative and competitive tasks, although limited, can also be applied to human-robot studies. These studies usually focus on joint attention using EEG in physically common space and shared responsibility (Hinz et al., 2021) or the relation with robots in teamwork (Lefkeli et al., 2021). This interaction however offers fewer ways in which robots and androids can interact with their environment since they are limited by their movement and lack of precision in which they manipulate objects.

Although there are differences in how particular forms of AI can influence social cognition, there are also factors that seem universal for every type of artificial agent as suggested by the current research:

- adaptability to human behavior in real-time taking into account the cultural background of the user, and enhancing acceptance (Alemi et al., 2021; Hauptman et al., 2023),
- humans’ beliefs and knowledge about the agent before the actual interaction, which may significantly influence their perception of its behavior (Wykowska et al., 2014; Henschel et al., 2020),
- easily interpretable and transparent social cues manifested by artificial agents (Banks, 2020; Jorge et al., 2024),
- usability and behavior appropriate for its role, which may be taken from user experience studies. Although user experience studies differ from those conducted in the fields of neuroscience and psychology, these types of studies are essential to understanding how the use of an artificial agent will impact social, cognitive, and emotional elements of human-agent interaction (Weiss et al., 2009; Silva et al., 2023),
- knowledge about the source of the agent’s behavior. Depending on whatever agent’s behavior is accompanied by the user’s input or, in the case of research studies, whatever participant is convinced that the agent is autonomous when in reality it is controlled by a human (usually referred to as the Wizard of Os method) (de Melo et al., 2013), the human’s stance and beliefs about the mental state of the agent may differ (Yu et al., 2023),
- beliefs about the moral stature and virtuous characteristics of the agent (Maninger and Shank, 2022; Bonnefon et al., 2024; Fortuna et al., 2024).

One prominent example of shared modalities is Replika, an AI companion designed for personalized social interaction. It can establish emotional bonds with users, particularly during stressful periods such as the COVID-19 pandemic (Trothen, 2022). Users often view Replika as a source of emotional support and psychological comfort, attributing human-like qualities to the avatar despite its cartoon-like appearance. Unlike traditional avatars, Replika offers a more immersive, customizable digital presence that promotes a deeper sense of connection. However, these attachments can sometimes lead to addictive behaviors and harm real-world relationships (Yuan et al., 2024). While Replika’s avatar-like qualities contribute to mental health benefits, these findings raise ethical concerns regarding its potential influence on users’ social, cognitive, and emotional well-being (Xie and Pentina, 2022). Replika can serve as an example of blurring boundaries between chatbots and avatars with common characteristics of both like text-based and audio-based communication, social presence (thanks to virtual reality integration) and personalization of the appearance (cosmetics and body and face changes).

Furthermore, AI companionship is increasingly being explored through the concept of Companionship Development Quality (CDQ), which defines the effectiveness of AI in fostering deep, meaningful, and lasting relationships with users (Chaturvedi et al., 2024). AI companions (ACs) are designed to integrate conversational, functional, and emotional capabilities to sustain user engagement. Conversational capabilities allow ACs to maintain natural, context-aware conversations, remembering past interactions to make discussions feel personalized. Functional capabilities enable ACs to assist users in practical tasks such as setting reminders, booking appointments, or controlling smart home devices, as seen in digital assistants like Alexa and Siri. Emotional capabilities include recognizing and responding to human emotions, facilitating social bonding, and reducing loneliness, exemplified by AI companions like Replika and Microsoft’s Xiaoice. Research suggests that AI designed with only functional or emotional traits tends to lose user engagement over time, leading to interaction decline. To avoid this, AI systems must balance all three capabilities, preventing users from falling into an uncanny valley where prolonged interaction leads to discomfort or loss of trust. AI companions that successfully integrate these capabilities can foster long-term human-AI relationships, enhancing emotional support, engagement, and usability. These systems along with their capabilities can be implemented to both physical representations of agents like robots but also to virtual entities like voice assistants which may have potential to integrate common features along different representations of AI.

Understanding the variables above is crucial for designing AI that fosters positive social interactions. However, another significant factor influencing human-AI interaction is the phenomenon known as the uncanny valley, which describes the discomfort people feel when interacting with agents that appear almost, but not entirely, human. Exploring this concept, and how it relates to different forms of artificial agents, can provide valuable insights into creating artificial agents that are both effective and comfortable for users.

### 4.2 Uncanny Valley

The uncanny valley describes the discomfort that arises when interacting with humanoid robots whose appearance closely resembles humans but falls short of full realism (Mori et al., 2012; Zhang et al., 2020). This phenomenon affects the perception of robots as sentient beings capable of feeling and decision-making. A meta-analysis of factors influencing the uncanny valley effect identified variables such as morphing faces to better match natural facial muscle movements, mismatched facial features, distorted biological movements, realism rendering, depictions of various characters, distorted or synthetic voices resembling androids, and human responses like emotions (disgust, fear) and esthetic feelings (symmetry, wrinkles; Diel et al., 2022). Designing improved AI through virtual agents, humanoid robots, and androids requires multidisciplinary collaboration among engineers, IT specialists, neuroscientists, cognitive scientists, and psychologists (MacDorman and Ishiguro, 2006). AI which is designed to serve as conversational partners, therapists, or tools to study social interactions cannot evoke any potential feeling of eeriness. For instance, androids can test theories about human interaction and brain functions in mediating communication. Failure to elicit appropriate social responses risks triggering the uncanny valley effect. This effect can be mitigated by designing AI suited to specific tasks and behaviors, such as a “nursebot” for hospital patients and the elderly. The uncanny valley effect can happen both on a psychological and neural level. Observing human-human interaction activates the left temporoparietal junction (one of the areas responsible for mentalization) more compared to observing human-robot interaction. In contrast, human-robot interactions activate the ventromedial prefrontal cortex and precuneus—areas associated with feelings of eeriness (Wang and Quadflieg, 2015).

Perceptions of robots’ capacities also affect feelings of eeriness in humans. Robots perceived as capable of experience (feeling emotions) elicit stronger feelings of eeriness compared to those seen as agents or mere tools. This effect is moderated in contexts where emotional sensitivity is valued, such as nursing, reducing the eeriness of experienced robots (Stein et al., 2020). The Uncanny Valley effect can also be measured outside of laboratory settings by analyzing what people think about robots on the internet (Ratajczyk, 2022). In one of those studies, Ratajczyk and team tried to address some issues in uncanny valley studies, including inconsistent similarity assessments, a focus on visual stimuli, and challenges in evoking genuine emotions in laboratory settings. Natural language processing was used to analyze YouTube comments on robot videos in social contexts. This method captured more authentic emotional reactions, revealing that human-like robots frequently triggered terms associated with uncanniness, with human-like robots often eliciting negative emotions. The analysis showed a relationship between facial features, sentiment, and horror, with words like “scary” and “terrifying” being most indicative of the uncanny valley effect. Interestingly, human resemblance did not correlate with pleasure or attractiveness, and smaller robots were perceived more positively, often viewed as toys. Additionally, the anticipated threat perception of larger robots was not confirmed.

Using the internet as a natural environment for studying the social perception of artificial agents also has its place in examining virtual influencers (VI). Highly anthropomorphized VIs—those with realistic human-like features—tend to elicit greater feelings of unease and uncanniness in users, potentially undermining their effectiveness as brand endorsers. This aligns with Mori’s uncanny valley theory, where near-human entities provoke discomfort due to their almost-but-not-quite-real appearance. Additionally, social cues, such as the inclusion of real human counterparts (like publicizing human-like activities like going out for a concert, or having coffee) in the VI’s content, can moderate this effect, making highly anthropomorphic VIs more acceptable to consumers by reinforcing a sense of familiarity and relatability (Creasey and Vázquez Anido, 2020; Gutuleac et al., 2024). Virtual influencers however do not exist only in the human-like form. Some of them take on the form of cartoon-like characters. Some studies suggest that in the case of cartoon-like characters, the feeling of eeriness may be lower compared to human-like influencers and cartoon-like may receive more positive reactions (indicated by the number of likes and the emotional tone of comments) compared to human-like influencers (Arsenyan and Mirowska, 2021a). This may be caused by doubt and skepticism about the human-like influencer’s authenticity, which is not present in interactions with the more stylized cartoon-like character. The level of distrust toward virtual influencers, similar to the case of chatbots, might be reduced by providing users with knowledge about the artificial nature of the avatar (De Brito Silva et al., 2022).

The factors of Uncanny Valley are associated mainly with visual cues, while the feeling of eeriness does not have to be necessarily limited to one modality. Chatbots can be interacted with either by typing or speaking with them and as artificial agents that also can play a role in healthcare settings (Ayers et al., 2023), they should also be studied in terms of potential negative/unnerving feelings toward them. Unlike robots but similar to virtual influencers, the research regarding the feeling of eeriness caused by interaction with chatbots is fairly new, mainly because of the fairly recent and fast evolution of large language models. Studies show that the uncanny valley effect may be triggered when chatbots impersonate the real person even when being empathetic and social (Skjuve et al., 2019; Park et al., 2023) but the feeling of eeriness does not appear in the same scenario when claiming full identity disclosure (in the sense that chatbot openly claims that it works based on large language model). Studies comparing communication with speech-based chatbots and text-based chatbots are lacking since most of the studies examine voice perception already implemented in the avatars (Song and Shin, 2024; Rao Hill and Troshani, 2024). These studies suggest that users respond more positively when expressive words and prosody are balanced with the avatar’s animation rather than overly animated, suggesting that subtle emotional cues in speech are preferable for a positive user experience without inducing uncanniness (Zhu et al., 2022). Other studies indicate that users experience more discomfort, negative affect, and psychophysiological arousal, such as increased heart rate and muscle tension when interacting with the animated avatar chatbot compared to the simpler text-based version agent (Ciechanowski et al., 2018) which creates a problem to establish whatever this effect is observed because of the voice features compared to the face features.

**Figure 1:**
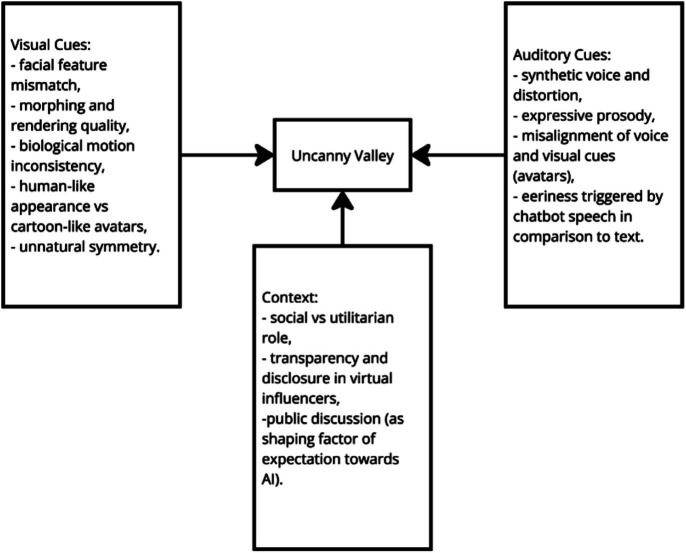
Diagram presenting how different types of cues are related to the Uncanny Valley effect. Particular cues will influence the interaction between humans and artificial agent depending of the modality (in example expressive prosody will increase the effect of Uncanny Valley toward voice-based assistants).

The smaller portion of the studies, focusing mostly on the speech, suggest that emotionally expressive prosody—such as varied pitch and enthusiastic interjections—significantly enhances user engagement and perceived human-likeness, but can also trigger discomfort when overly human-like traits lead to an uncanny valley effect (Krauter, 2024). Krauter conducted an extensive analysis regarding the factors associated with an uncanny valley in chatbots including the aforementioned expressive prosody. His work however can also be adjusted to other virtual agents like robots and avatars while trying design studies that compare these forms between the same social tasks. This will allow future researchers to establish what elements of uncanniness are present in particular forms of AI and how they can be adjusted to meet social cognition needs (Figure 1).

The uncanny valley affects how people perceive artificial agents’ capacities for emotions and decision-making, especially in contexts where emotional sensitivity is valued, such as healthcare. Studies mentioned above indicate that human-robot interaction activates brain areas associated with eeriness, while human-human interaction engages regions linked to natural social processes. Robots perceived as capable of experiencing emotions elicit stronger feelings of eeriness, though this is reduced in emotionally sensitive roles. For virtual influencers, human-like designs may evoke skepticism about authenticity, whereas cartoon-like designs generate higher engagement and positive affect. This pattern suggests that anthropomorphism should be balanced with clear identity disclosure to reduce unease. Similarly, chatbots may induce uncanny valley effects when mimicking humans, particularly in speech-based interactions, though this can be mitigated by openly acknowledging their AI nature. Subtle emotional cues in speech, rather than overly human-like traits, enhance user experiences without triggering discomfort. Addressing these factors to the proper form of the agent will help design artificial agents that balance human likeness with user comfort, fostering positive social interactions while meeting social cognition needs. One of the possible solutions to reduce the feeling of eeriness and make the interaction between humans and AI more social and natural might be associated with a more personalized approach in designing robots, avatars, or chatbots. This will be the topic of the next section.

## 5 Conclusion

The growing study of human-artificial agent interaction underscores the increasing significance of AI design in shaping social experiences and cognitive processes. As artificial agents such as humanoid robots, virtual avatars, and chatbots continue integrating into social, therapeutic, and professional environments, their design and behavioral adaptability profoundly influence human perception, engagement, and emotional connection. We presented the mechanisms underlying these interactions, demonstrating that the form, functionality, and perceived mental capacities of artificial agents directly impact the depth and quality of human-AI relationships.

From the design perspective, human-artificial agent interaction is shaped by an artificial agent’s embodiment, expressiveness, and perceived autonomy. Humanoid robots benefit from physical presence and non-verbal cues but risk triggering the uncanny valley effect. Virtual avatars offer flexible social representation in digital environments but lack the nuances of face-to-face interaction. Chatbots, engaging primarily through language, enhance accessibility yet lack physical expressiveness. Despite these limitations, conversational AI continues to improve in eliciting empathy and fostering engagement while also synergizing with both robots and avatars.

Physical appearance and visualization significantly influence human attribution of mental states to AI. More human-like agents enhance mind attribution, trust, and social presence, affecting whether they are seen as tools, companions, or social peers. Engagement is driven by social and emotional mechanisms, such as trust and expectation alignment, reinforcing AI as a social entity.

The uncanny valley remains a challenge in AI design, where human-like features must be balanced to avoid discomfort. Avatars and chatbots, with more controlled anthropomorphism, integrate more seamlessly into social settings without triggering unease. Advancing AI social design will require interdisciplinary collaboration to foster meaningful, trustworthy, and emotionally intelligent interactions.

Future research must continue to examine the ethical and psychological implications of human-AI interaction, particularly in contexts where artificial agents serve roles traditionally reserved for human counterparts. Furthermore, interdisciplinary efforts involving cognitive science, psychology, robotics, and ethics are necessary to develop AI that is not only technologically proficient but also socially attuned to human expectations and needs.

In summary, the design of artificial agents plays a foundational role in shaping human-AI social interactions. By carefully considering embodiment, appearance, behavioral transparency, and adaptability, developers can create AI systems that foster trust, social connection, and emotional engagement. As AI technology advances, the key to successful human-AI interaction will lie in crafting agents that align with human social and emotional processes while respecting the boundaries of what is natural and what is artificial. This ongoing evolution demands a nuanced understanding of both technological innovation and the fundamental principles governing human social behavior (Table 1).

**Table 1:** Overview of selected papers investigating social factors relative to AI design type.

Authors	Type of AI	Investigated social factors	Type of interaction	Findings
Felnhofer et al. (2024)	Avatar	Social presence, agency perception, evaluation	Observing and interacting with virtual humans in immersive VR	Avatars were rated higher in social presence and evaluation than AI agents, but behavioral responses did not significantly differ. Social presence was more pronounced in neutral tasks compared to negative ones. The study suggests that higher-order responses (e.g., evaluation, presence) are influenced by perceived agency, while automatic behaviors remain unchanged.
Fraser et al. (2024)	Avatar	Perceived realism, enjoyment	Disclosing positive and negative experiences in VR	Avatars with high human resemblance and graphical resolution were perceived as the most realistic, but both cartoon and high-realism avatars were rated equally enjoyable. Standard avatars, commonly used in social VR, were rated least enjoyable, suggesting that enhancing graphical realism may improve social VR experiences.
Lim et al. (2024)	Avatar	Social influence, presence, gender matching	Conversing with a VR-embodied conversational agent (VR-ECA) about health	VR-ECAs enhanced perceived presence and social connection compared to text-based chatbots. Gender matching did not significantly impact likeability, but opposite-gender pairings increased gaze duration and slightly influenced healthy snack selection. Female participants rated VR-ECAs more favorably than male participants.
Woo et al. (2024)	Avatar	Adaptation, engagement, cognitive change	Engaging in cognitive behavior therapy (CBT) with an adaptive virtual agent	Adaptive virtual agents that adjust facial expressions and head movements based on users’ behavior enhanced engagement and effectiveness in cognitive behavior therapy. Users perceived adaptive agents as more human-like and reported greater cognitive change and anxiety reduction. However, non-adaptive agents or those with mismatched behaviors negatively impacted user experience.
Arsenyan and Mirowska (2021b)	Avatar	Uncanny Valley, social media engagement, authenticity	Analysis of human reactions to virtual influencers on Instagram	Human-like virtual influencers received significantly fewer positive reactions compared to human and anime-like influencers, supporting the Uncanny Valley hypothesis. The study highlights authenticity concerns and social identity effects when interacting with virtual agents in publicly visible online networks.
Shin (2020)	Avatar	Intimacy, emotional engagement, social media interaction	Analysis of user interactions with virtual agents on Instagram	Users are more likely to engage with virtual agents when they express emotions in their posts. Emotional expression and relationships between virtual agents attract higher numbers of likes and comments. The study highlights the importance of emotional cues in fostering social engagement with AI entities on social media.
Von Der Pütten and Krämer (2010)	Avatar	Social presence, behavioral realism, agency perception	Observing and interacting with virtual agents and avatars	Participants’ beliefs about interacting with an avatar or an agent had minimal influence on their social responses, but higher behavioral realism significantly increased perceived presence and engagement. The findings support the Ethopoeia concept, suggesting that social cues, rather than perceived agency, drive human social responses to AI.
Figueroa-Torres (2025)	Chatbot	Social dimensions of chatbot technology	Theoretical analysis of chatbot roles in science, commerce, and personal life	Chatbots function across three dimensions: as scientific objects, commercial commodities, and agents of intimate interaction. Their roles extend beyond mere functionality, shaping and being shaped by society. The study emphasizes the importance of understanding chatbot technology through a sociotechnical lens rather than a purely technological progression.
Ali F. et al. (2024)	Chatbot	Social anxiety, fear of rejection, compulsive chat	Frequent interaction with a social chatbot (Xiaoice)	Socially anxious individuals with a fear of negative evaluation and rejection are more likely to engage in compulsive chatbot interactions. Fear of unavailability of human social connections further strengthens this behavior. The study highlights how social chatbots may serve as coping mechanisms for anxiety but also risk fostering dependency.
Bialkova (2024)	Chatbot	Functionality, interactivity, enjoyment, satisfaction	Survey-based evaluation of chatbot user experience	Information quality, accuracy, and competence were key factors in chatbot functionality. Personal care and social presence enhanced user enjoyment. Poor chatbot performance in these areas resulted in low satisfaction, highlighting the need for optimized chatbot design to meet user expectations.
Yu and Zhao (2024)	Chatbot	Perceived warmth, competence, service satisfaction	Experimental study on emoji usage in chatbot interactions	Emojis enhance the perceived warmth of chatbots, increasing service satisfaction, but do not improve perceptions of competence. The effect is stronger for hedonic chatbots and pre-programmed bots compared to highly autonomous ones. The study highlights the role of emojis as social cues in chatbot communication.
Pan et al. (2024)	Chatbot	Uncertainty, emotional attachment, relational dynamics	Analysis of user discussions in an online chatbot community	Users experience four key uncertainties when forming relationships with social chatbots: technical, relational, ontological, and sexual uncertainty. Relational uncertainty was the most common, often leading to emotional attachment and mixed feelings about AI companionship. Some users embraced unpredictability, while others felt discomfort or confusion.
Kang and Kang (2024)	Chatbot	Self-disclosure, companionship, anthropomorphism	Engaging in a counseling session with a chatbot	The chatbot’s anthropomorphic features, including gender, personality, and visual interface cues, influenced user self-disclosure and companionship. Users disclosed less when the chatbot had a visual interface cue, especially when it had an introverted personality. Participants felt a stronger companionship with chatbots of the opposite gender. The study highlights the importance of tailoring chatbot design to user characteristics.
Rheu et al. (2024)	Chatbot	Expectancy violation, trust, emotional validation	Engaging in a support-focused conversation with a chatbot	Chatbots that provided contingent, personalized feedback were evaluated more positively than those that gave generic responses. When an “expert” chatbot failed to provide contingent feedback, it led to more negative evaluations (negative expectancy violation). However, a non-expert chatbot that exceeded expectations by offering contingent feedback was evaluated favorably (positive expectancy violation). The study highlights the importance of meeting user expectations in chatbot interactions.
Pekçetin et al. (2024)	Robot	Mind perception, agency, experience attribution, generational differences	Observing live human and robot actors perform communicative and non-communicative actions	Real-time implicit and explicit measurements revealed that people attribute higher agency and experience to humans than robots. Communicative actions increased mind perception more than non-communicative actions. Generational differences influenced responses, with younger participants attributing greater mental states to robots. Implicit and explicit results varied, suggesting different cognitive mechanisms behind mind perception in HRI.
Kamino et al. (2024)	Robot	Social bonding, interaction rituals, cultural integration	Ethnographic study of social robot communities in Japan	The study explores how users integrate robots into their social lives through recurring interaction rituals, such as meetups, co-ownership events, and daily routines. Companies facilitate social bonding by organizing events and designing robots with customizable features that promote user attachment. The findings highlight that robot sociality is not just a product of design but is actively constructed through human interactions in social networks and communities.
Ghiglino et al. (2023)	Robot	Intentional stance, social bonding, interaction variability	Engaging with the iCub robot in different interaction scenarios	The study analyzed how different levels of interaction with the iCub robot influenced participants’ attribution of intentional states to the robot. When the robot exhibited highly human-like, contingent behaviors, participants were more likely to adopt a mentalistic stance, interpreting its actions as intentional. However, in low-interactivity scenarios, participants tended to maintain a mechanistic perspective. The study suggests that deeper social engagement with robots enhances perceived intentionality.
Karaduman et al. (2023)	Robot	Empathy, pain perception, emotional vs. physical pain	Watching videos of humans and robots experiencing pain and rating perceived intensity	Participants attributed significantly more pain to humans than to robots in both physical and emotional scenarios. Emotional pain ratings varied depending on whether the pain source was an object or a person, whereas physical pain ratings were stable across conditions. The study highlights a persistent gap in empathy toward non-biological agents.
Spisak and Indurkhya (2023)	Robot	Social exclusion, trust, team dynamics	Cooperating with the Nao robot in a bomb defusal task	The study examined social exclusion in human-robot teams. When the robot favored one participant over another, the discriminated participant reported a stronger sense of exclusion but did not significantly change their mood or attitude toward the robot. The findings highlight the potential social implications of biased robot behavior in group interactions.
Churamani and Howard (2022)	Robot	Affective learning, adaptability, negotiation strategies	Interacting with a social robot (NICO) in a negotiation game	The study introduces an affect-driven learning framework for robots, where the NICO robot adapts its negotiation strategy based on users’ affective responses. Results show that robots with patient and high-arousal affective cores negotiate longer and retain persistence, whereas those with impatient and low-arousal dispositions are perceived as more generous and altruistic. The findings highlight the importance of affective appraisal in human-robot interaction and adaptive behavior learning.
Jacobs and Turner (2022)	Robot	Mind perception, agency, experience attribution	Rating agency and experience of real and fictional robots	The study found significant variation in how people attribute agency (capacity to act) and experience (capacity to feel) to different robots. While robots were rated lower than humans, some real robots, like Sophia and Atlas, received higher attributions of experience than digital assistants like Siri or Alexa. Younger participants attributed higher levels of agency and experience to robots, suggesting a generational shift in AI perception.

## Footnotes

^1^In the context of artificial agents, the ToM test evaluates an AI system’s ability to infer and respond to human mental states, such as beliefs, intentions, and emotions, simulating aspects of human social cognition.

^2^The Turing Test is a benchmark for evaluating an artificial agent’s ability to exhibit human-like intelligence by engaging in natural language conversations indistinguishable from those of a human. In this test, a human evaluator interacts with both an AI and a human without knowing which is which, and if the AI can convincingly mimic human responses, it is considered to have passed the test, demonstrating a form of machine intelligence.

^3^A Faux Pas Test assesses an AI system’s ability to recognize socially inappropriate or unintended offensive remarks in a conversation, demonstrating an understanding of social norms and implicit meanings. This test evaluates whether an AI can detect when a statement might embarrass or offend someone, requiring it to infer the mental states of the speaker and listener.

^4^The Proteus effect explains how individuals’ behaviors and attitudes align with the characteristics of their avatars in virtual environments, such as appearance or perceived traits, influencing real-world interactions and actions. For example, a person using a taller avatar in a virtual negotiation may become more assertive and confident compared to when using a shorter avatar.

^5^The metaverse is a virtual shared space that emerges from the merging of enhanced physical and digital realities. It serves as an interconnected and immersive digital ecosystem where users engage in real-time interactions through avatars, participating in a wide range of activities such as socializing, working, gaming, and trading. Source: https://about.meta.com/metaverse/.

^6^An NPC (non-playable character) is a character in a game or simulation controlled by the system, not the player, often serving roles like guides, adversaries, or background figures to enrich the narrative or gameplay experience.

^7^Machine learning empowers computational systems to acquire knowledge and enhance their performance through experience and data analysis, all while operating without the need for explicit programming directives. Source: https://www.ibm.com/think/topics/machine-learning.

^8^Natural language processing aims to help computers comprehend, interpret, and produce human language in a significant manner. Source: https://www.ibm.com/think/topics/natural-language-processing.

^9^Source: https://chat.mistral.ai/chat.

^10^Source: https://www.deepseek.com/.

^11^Appropriate animation can be set thanks to the rigging system in some of the avatars. A rig is a digital skeleton used in 3D modeling to define how an avatar’s structure moves, enabling the implementation of animations by controlling its joints and limbs.
